# Structure-Function Analysis of Human TYW2 Enzyme Required for the Biosynthesis of a Highly Modified Wybutosine (yW) Base in Phenylalanine-tRNA

**DOI:** 10.1371/journal.pone.0039297

**Published:** 2012-06-28

**Authors:** Virginia Rodriguez, Sona Vasudevan, Akiko Noma, Bradley A. Carlson, Jeffrey E. Green, Tsutomu Suzuki, Settara C. Chandrasekharappa

**Affiliations:** 1 Cancer Genetics Branch, National Human Genome Research Institute, Bethesda, Maryland, United States of America; 2 Department of Biochemistry and Molecular Cellular Biology, Georgetown University Medical Center, Washington, District of Columbia, United States of America; 3 Department of Chemistry and Biotechnology, Graduate School of Engineering, The University of Tokyo, Tokyo, Japan; 4 Laboratory of Cancer Prevention, National Cancer Institute National Institutes of Health, Bethesda, Maryland, United States of America; 5 Laboratory of Cancer Biology and Genetics, National Cancer Institute, National Institutes of Health, Bethesda, Maryland, United States of America; Max-Planck-Institute for Terrestrial Microbiology, Germany

## Abstract

Posttranscriptional modifications are critical for structure and function of tRNAs. Wybutosine (yW) and its derivatives are hyper-modified guanosines found at the position 37 of eukaryotic and archaeal tRNA^Phe^. TYW2 is an enzyme that catalyzes α-amino-α-carboxypropyl transfer activity at the third step of yW biogenesis. Using complementation of a ΔTYW2 strain, we demonstrate here that human TYW2 (hTYW2) is active in yeast and can synthesize the yW of yeast tRNA^Phe^. Structure-guided analysis identified several conserved residues in hTYW2 that interact with S-adenosyl-methionine (AdoMet), and mutation studies revealed that K225 and E265 are critical residues for the enzymatic activity. We previously reported that the human TYW2 is overexpressed in breast cancer. However, no difference in the tRNA^Phe^ modification status was observed in either normal mouse tissue or a mouse tumor model that overexpresses Tyw2, indicating that hTYW2 may have a role in tumorigenesis unrelated to yW biogenesis.

## Introduction

**Figure 1:**
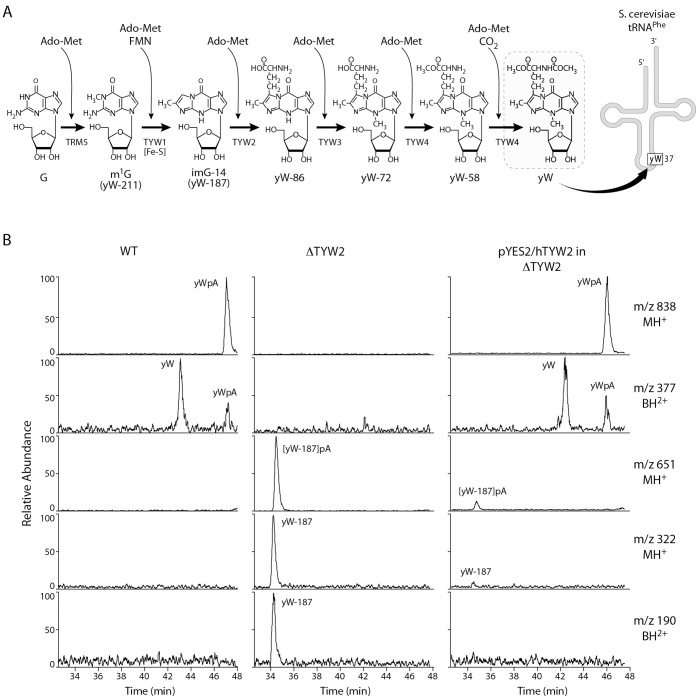
Human TYW2 has the same enzyme activity as its yeast counterpart in Wybutosine (yW) biosynthesis. (A) Wybutosine (yW) biosynthetic pathway in yeast. The yW base is located adjacent to the anticodon in tRNA^Phe^. The pathway for yW biosynthesis in yeast has been described earlier [1]. (B) LC/MS analysis of nuclease P1 digested tRNA-Phe obtained from wild type (WT), the TYW2 deletion strain (ΔTYW2), and the deletion strain transformed with pYES2/hTYW2 (pYES2/hTYW2 in ΔTYW2). The panels show mass chromatograms detecting MH+ (m/z 838) of yWpA, BH2+ (m/z 377) of yW, MH+ (m/z 651) of yW-187pA, MH+ (m/z 322) of yW-187 and BH2+ (m/z 190) of yW-187, respectively.

Though over 100 modified bases have been characterized in tRNAs, the biosynthetic pathways leading to many modifications in mammalian tRNAs are not well understood. Wybutosine (yW) is one of the highly modified bases, located at position 37 in tRNA^Phe^, and its biosynthetic pathway, including characterization of four novel TYW enzymes (tRNA-yW synthesizing proteins), has now been elucidated in yeast [1]. In yeast, the biosynthesis of yW consists of a six-step process involving five different enzymes: TRM5, TYW1, TYW2, TYW3, TYW4 (Figure 1A) [1], and each step is mediated by the binding of S-adenosyl-methoinine (AdoMet). In the first step of the reaction, the G37 base of tRNA^Phe^ is methylated by TRM5, a methyltransferase, using AdoMet as the methyl group donor. Step two is the formation of a tricyclic ring catalyzed by TYW1, an iron-cluster protein. Step three is mediated by a transferase, TYW2, which transfers the bulky α-amino-α-carboxypropyl (acp) group from AdoMet to the side-chain at C-7 position of yW-187 to produce yW-86. Step four is the methylation of the N-4 position of yW-86, by TYW3, to yield yW-72. Steps five and six are mediated by TYW4, a carboxymethyltransferase. Methylation of the α-carboxy group of yW-72 forms yW-58, then methoxycarbonylation of the α-amino group of yW-58 produces the fully modified yW base. The human gene, initially known as *TRMT12* and *TRM12*, was renamed *TYW2* based on its sequence homology to the yeast *TYW2* gene. We wanted to explore whether the human TYW2 provides a similar enzymatic activity to its yeast counterpart in yW biosynthesis.

The yW base in yeast tRNA^Phe^ was initially described nearly four decades ago [2], and is found exclusively in tRNA^Phe^ from Eukarya and Archaea [3]. The guanosine (G) at position 37, immediately 3′ to the anticodon in tRNA^Phe^, undergoes posttranscriptional modification to yW. The yW base stabilizes the codon-anticodon interaction and functions to maintain the correct reading frame [4]. Early on, it was observed that tRNA^Phe^ from rat and mouse tumors, unlike normal tissues, did not carry the fully modified yW base [5]. Later, it was determined that the under-modification of the yW base in tRNA^Phe^ could cause –1 frameshifting during translation [6]. Previously, we demonstrated that human *TYW2* (*hTYW2*) was amplified and overexpressed in breast cancer. High-density BAC arrays revealed amplification of an ∼1 Mb region on chromosome 8 in several breast cancer cell lines, and *hTYW2*, located within this genomic region, showed the highest expression across all the cell lines. RNA from 30 breast tumors was examined and *hTYW2* was found to be expressed >2 fold in 87% of the tumors [7]. Therefore, it was of interest to evaluate whether overexpression of the *hTYW2* disrupted the wybutosine pathway in the mammary tumor cells.

In this study, we identify a mouse mammary tumor model that overexpresses *Tyw2* and explore whether the biosynthesis of yW base is compromised in tRNA^Phe^ from the tumors. We demonstrate in an *in vivo* system that the biological function of human TYW2 in the posttranscriptional modification of tRNA^phe^, is similar to that of its yeast homolog. Additionally, based on our observation that human TYW*2* catalyzes the transfer of an acp group from AdoMet, implying a crucial role in the biosynthesis of yW, we use a homology model to predict the critical resides for enzymatic activity and follow up with mutagenesis studies to provide experimental verification.

## Materials and Methods

### Mouse Mammary Tissue

Normal mouse mammary tissue was obtained from pregnant FVB/N mice at about 18 days of gestation. Mammary tumor tissue was obtained from mice heterozygous for the C3 (1)/SV40-T/t-antigen [C3 (1)/Tag] transgene in the FVB/N background as previously described [8]. Mice were euthanized at about 5 months of age when tumors were 1–2 cm in diameter. Mammary tumors were also extracted from MMTV-Her2/neu [9] and MMTV-PymT [10] transgenic mice when they developed generally between 5 and 7 months of age.

### cDNA Preparation and Quantitative PCR

Total RNA was extracted with phenol-chloroform (TRIzol reagent, Invitrogen), treated with DNase, and the quality of the RNA was assessed using the 2100 Bioanalyzer (Agilent Technologies, Cedar Creek, TX). cDNA was prepared using the SuperScript First strand Synthesis kit (Invitrogen). The 2-ΔΔCt method was used to determine the Tyw12 gene expression and represented as fold changes relative to that of the normal mouse mammary sample [11]. ß2M gene expression served as the internal reference and quantitative PCR (RT-qPCR), was performed using the Fast SYBR Green kit (Applied Biosystems, Foster City, CA). Mouse Tyw2 primers: GTGCCAACTTAGGGCTTGAG and CCTCGGGTAATGAGAAACCA, and ßB2M primers: GATCATATGCCAAACCCTCTG and TGGGGGTGAGAATTGCTAAG.

### Plasmids

The human hTYW2 expression plasmid was obtained from Origene (Rockville, MD) (cat# TC113870). The coding region of hTYW2 was PCR amplified and cloned into the yeast (pYES2) vector by standard cloning techniques, and the construct was verified by sequencing.

### Yeast Complementation Test

*Saccharomyces cerevisiae* ΔTYW2 gene deletion strain was obtained from EUROSCARF: the Y10571 strain (BY4742 (*Matα; his3Δ1; leu2Δ0; lys2Δ0; ura3Δ0*), *YML005w::kanmx4*) was transformed by pYES2/hTYW2. The transformant was cultivated overnight in SC-ura/glucose media (0.67% yeast nitrogen base without amino acids, 0.5% casamino acid, and 2% glucose supplemented by auxotrophic nutrients as specified without uracil), and then diluted into SC-ura/raffinose media (0.67% yeast nitrogen base without amino acids, 0.5% casamino acid, and 2% raffinose, supplemented by auxotrophic nutrients as specified without uracil) at a starting OD_600_ ≈ 0.4. Protein was induced by culturing in YPG media (2% peptone, 1% yeast extract and 2% galactose) for 20 hours. Total tRNA from each strain was extracted, and their modified nucleosides were analyzed by LC/MS.

### Mass Spectroscopy

Total tRNA was isolated from total RNA from each strain on 10% polyacrylamide gels containing 7 M urea. To analyze RNA nucleosides, 20 µg of total tRNA was digested to nucleosides with nuclease P1 (Yamasa, Salem, OR) and bacterial alkaline phosphatase derived from *Escherichia coli* strain C75 (BAP.C75), (Takara Mountain View, CA) for 3 h at 37°C, and analyzed by LC/MS using ion trap mass spectrometry as described previously [1]. Nucleosides were separated by an ODS reverse-phase column (Intersil ODS3 5 µm, 2.1×250 mm, GL Science) using an HP1100 liquid chromatography system (Agilent Technologies, Palo Alto, CA). The solvent consisted of 0.1% acetonitrile in 5 mM NH_4_OAc (pH 5.3) (Solvent A) and 60% acetonitrile in H_2_O (Solvent B) in the following gradients: 1–35% B in 0–35 min, 35–99% B in 35–40 min, 99% B in 40–45 min, 99–1% B in 50–50.1 min and 1% B in 50.1–60 min. The chromatographic effluent was directly conducted to the electrospray ionization (ESI) source to ionize the separated nucleosides, which were analyzed on a LCQ DUO ion trap mass spectrometer (Thermo Fisher Scientific, Pittsburgh, PA). The mass spectrometer was operated with a spray voltage of 5 kV and a capillary temperature of 245°C. The sheath gas flow rate was 95 arb, auxiliary gas flow rate was 5 arb. Positive ions were scanned over an m/z range of 103 to 900.

### tRNA Extraction, Labeling, and Fractionation

Total tRNA was extracted from 1 gram of either normal or tumor mouse mammary tissue [12], aminoacylated with [^3^H]-phenylalanine and 19 unlabeled amino acids under limiting tRNA conditions in the presence of rabbit reticulocyte synthetases [13]. The resulting aminoacylated tRNA was fractionated on a RPC-5 column [14] as described previously [13]. Total calf liver tRNA was purchased from Sigma (St. Louis, MO) and aminoacylated with [^14^C]-phenylalanine as described above and co-chromatographed with [^3^H]-phenylalanine labeled normal or tumor mouse tRNA.

### Northern Blot Analysis

Total tRNA was quantified, and 0.8 µg of each sample was run on a 15% TBE-Urea gel (Invitrogen, Carlsbad, CA), and transblotted onto a nylon membrane. The membrane was hybridized with a ^32^P-5′-end-labeled oligonucleotide that was complementary to the sequence 5′- TGCCGAAACCCGGGATCGAACCAGGGAC-3′ at the 3′ end of tRNA^Phe^ and the Northern blot was analyzed using a PhosphorImager. The blot was stripped and probed with a ^32^P-5′-end-labeled oligonucleotide that was complementary to the 20 nucleotides at the 3′ end of tRNA^Ser1^ that was used as an internal control, Ser [15].

### Bioinformatics and Structural Analysis

The protein information was obtained from the UniProt database (www.uniprot.org). Domain information was obtained from the Pfam database (http://pfam.sanger.ac.uk/). The structural information for the available homologous structures was obtained from the Protein Data Bank, PDB (www.rcsb.org). Topological information was obtained from PDBSum (http://www.ebi.ac.uk/pdbsum/) database [16]. Structure-guided alignment of the homologous sequences and structures was done using Cn3d tool implemented within the CDTree (http://www.ncbi.nlm.nih.gov/Structure/cdtree/cdtree.shtml) tool [17]. Single-linkage clustering was done using the CDTree tool. Protein family classification was done using the PIRSF classification system (pir.georgetown.edu). Classification of protein sequences or structures into families that reflect their ancestry is valuable in providing clues about a protein’s function. The PIRSF system built as a hierarchical structure was designed to provide a solid framework that enables functional annotation at various levels of hierarchy. This system clusters full-length proteins into homeomorphic families [18]. Proteins are assigned to the same PIRSF only if they share end-to-end similarity including similar domain architectures. The homologous sequences used to compute the tree were based on PIRSF family memberships. Homology modeling was done using the Swiss-pdb viewer [19]. The model was refined and minimized using the GROMOS986 potentials implemented in Swiss-pdb viewer.

## Results

Human TYW2 can substitute for the yeast enzyme in the biosynthesis of yW base in tRNA^Phe^ Sequence analysis shows that the human TYW2 protein (448 amino acids) shares 35% identity with the yeast TYW2 protein (462 amino acids), and the homology primarily resides in its transferase domain (amino acids 118–336). The N-terminal 117 residues and the C-terminal 112 residues of hTYW2 do not resemble any known domain. However, the former shows homology to TYW2 sequences from mammals and the latter is conserved even in sequences from plants and protozoans. To determine whether hTYW2 can replace the function of the yeast TYW2 *in vivo*, a yeast complementation test was performed. The h*TYW2* gene was cloned into the yeast vector pYES2, and the ΔTYW2 deletion strain, Y10571, was transformed by pYES2/hTYW2. Total tRNA was extracted from wild type, deletion and transformed yeast strains, and their modified nucleosides were analyzed by LC/MS (Figure 1B). Wybutosine (yW) was detected as the proton adduct form (MH+) of the dinucleotide yWpA (m/z 838, RT 46.8 min) and a peak for the protonated base fragment (BH_2_+) of yW (m/z 377) in the wild type strain (left panel). These peaks are absent in the ΔTYW2 strain (middle panel), but are detected when the deletion strain is transformed with pYES2/hTYW2 (right panel). Also, the intermediate yW-187pA (m/z 651, RT 34.5 min) shown in the middle panel is no longer present in the right panel indicating that yW-187pA has been converted to the fully modified yW base. This confirmed that the human hTYW2 protein catalyzes the third step in the biosynthesis of wybutosine and thus established a biological function of this protein in mammalian cells.

### Model Building and Sequence Analysis to Identify the Amino Acid Residues that are Critical for Enzymatic Activity in hTYW2

In order to identify critical and functionally important residues in hTYW2, analysis at various sequence and structure levels was carried out. Based on sequence analysis, the query protein Q53H54 (TYW2_HUMAN) is an AdoMet-dependent transferase with substrate specificity for tRNAs. The yeast and archaeal homologs of TYW2 have been shown to be transferases that transfer a α-amino-α-carboxypropyl group (acp) from AdoMet instead of a methyl group which is typical of AdoMet-dependent transferases [1], [20]. Domain analysis was carried out using the Pfam database [21], and hTYW2 belongs to PF02475 Met-10+ like-protein family. This family contains proteins involved in methyltransferase activity and proteins involved in methionine biosynthesis. The members of this family display about 40 different domain architectures with majority containing only the Met-10+ domain. Residues 118–336 of hTYW2 contain the transferase domain. We generated a homology model of hTYW2 based on the crystal structure of protein PH0793 (TYW2) from *Pyrococcus horikoshii* (PDB-ID: initially 2FRN, and then 3K6R) in order to locate important residues in the AdoMet binding pocket. Since the sequence homology between the archaeal TYW2 and hTYW2 was only 35%, the model was generated so not to miss any features specific to hTYW2. We re-computed the model using the crystal structure of the same (*Pyrococcus horikoshii* TYW2) protein in complex with AdoMet (PDB-ID: 3A25) [20], after it became available. The hTYW2 protein models based on the unbound and the AdoMet-bound *Pyrococcus horikoshii* TYW2 crystal structures were consistent with each other.

Analysis of 300 or so AdoMet bound structures (data not shown) shows that structures of methyltransferases belonging to Class I, in general, display two major strand arrangements 6754123 and 3214576 (with strand 7 anti-parallel to the remaining strands). The hTYW2 belongs to AdoMet-dependent methyltransferase SCOP (http://scop.mrc-lmb.cam.ac.uk/scop) fold with a topology consistent with Class I methyltransferases. The arrangement of beta strands is in the order 6754123 with strand 7 anti-parallel to rest of the strands as is typically seen in this fold. It belongs to the class of alpha/beta proteins as per SCOP classifications [22].

Family classification analysis using PIR classification system shows that hTYW2 belongs to PIRSF038667 homeomorphic family consisting specifically of mammals, while the archaeal and yeast homologs belong to PIRSF006525 and PIRSF038972 respectively. The PIRSF classification of proteins is based on end-to-end similarity and similar domain architectures. The average lengths of the members belonging to PIRSF038667, PIRSF06525 and PIRSF038972 are 437, 325 and 430 amino acids respectively. While the mammalian and yeast proteins have similar lengths, their sequence identity is only about 35%. Yeast TYW2 has diverged far away from its archaeal homologs with sequence identities of only 23%. It is interesting that although the sequences have diverged and fall into distinct families, the proteins have highly conserved structure and function. Single-linkage clustering analysis shows three distinct branches separating archaeal, fungal, and mammalian TYW2 proteins (Figure S1, Table S1).

**Figure 2:**
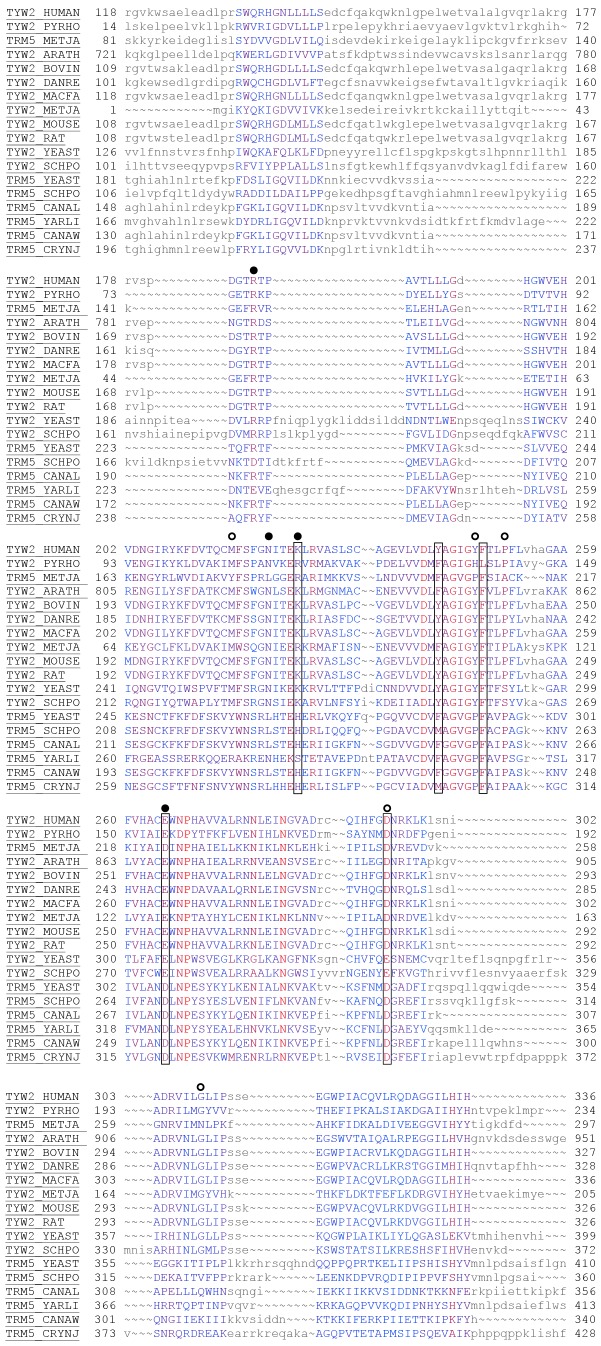
Structure-guided alignment of hTYW2 protein with its homologs. The alignment shows 18 sequences from the Swiss-Prot (reviewed, manually annotated) subset of the UniProtKB database (www.uniprot.org). The alignment of all 49 sequences in the UniProtKB database, both reviewed (Swiss-Prot) and un-reviewed (TrEMBL) sequences are presented as Figure S2. The alignment was created using the Cn3d tool. The color of the residues reflect the level of conservation with highly conserved residues in red to not conserved residues in blue, and the residues in smaller letters indicate regions of no conservation. This alignment shows only the transferase domain corresponding to residues 118–336 of hTYW2. The sequences are labeled using UniProtKB accessions. The boxes around the positions of five residues in TYW2 HUMAN (K225, Y242, F248, E265 and D293) are to indicate that they were chosen for mutagenesis studies. The circles indicate the nine residues in TYW2 PYRHO that were analyzed by mutagenesis, and those resulting in the severe inactivation of the enzyme activity (>90%) are with filled circles (from Umitsu et al Proc Natl Acad Sci U S A 106: 15616–15621). PYRHO: *Pyrococcus horikoshii*; METJA: *Methanocaldococcus jannaschii;* ARATH: *Arabidopsis thaliana*; BOVIN: *Bos taurus*; DANRE: *Danio rerio*; MACFA: *Macaca fascicularis*; METJA: *Methanocaldococcus jannaschii*; MOUSE: *Mus musculus*; RAT: *Rattus norvegicus*; YEAST: *Saccharomyces cerevisiae*; SCHPO: *Schizosaccharomyces pombe*; CANAL: *Candida albicans*; YARLI: *Yarrowia lipolytica* CANAW: *Candida albicans*; CRYNJ: *Cryptococcus neoformans.*

In order to identify key conserved residues in the AdoMet binding pocket for mutagenesis studies, a structure-guided alignment of structures and sequences from members of the three PIRSFs was done using Cn3d/CDtree tool. Since the conservation is only at the structural level, a structure-guided alignment rather than a traditional sequence alignment is more meaningful. An alignment (Figure S2, Table S1) was constructed with all the representative members from the three PIRSFs, a total of 49 sequences. Another alignment was constructed from 18 members within this set that have been annotated/reviewed in the UniProt Swiss-Prot section of the UniProtKB database and is presented in Figure 2. Based on the alignment and the evaluation of other Class I structures (data not presented), candidates that make H-bond interactions with the AdoMet moiety were chosen for mutagenesis experiments: K225, Y242 (Y242 displays a stacking interaction with the adenosine moiety), F248, E265, and D293.

### Mutagenesis of the Critical Residues in hTYW2 and Evaluation of its Consequence on yW Biosynthesis

**Figure 3:**
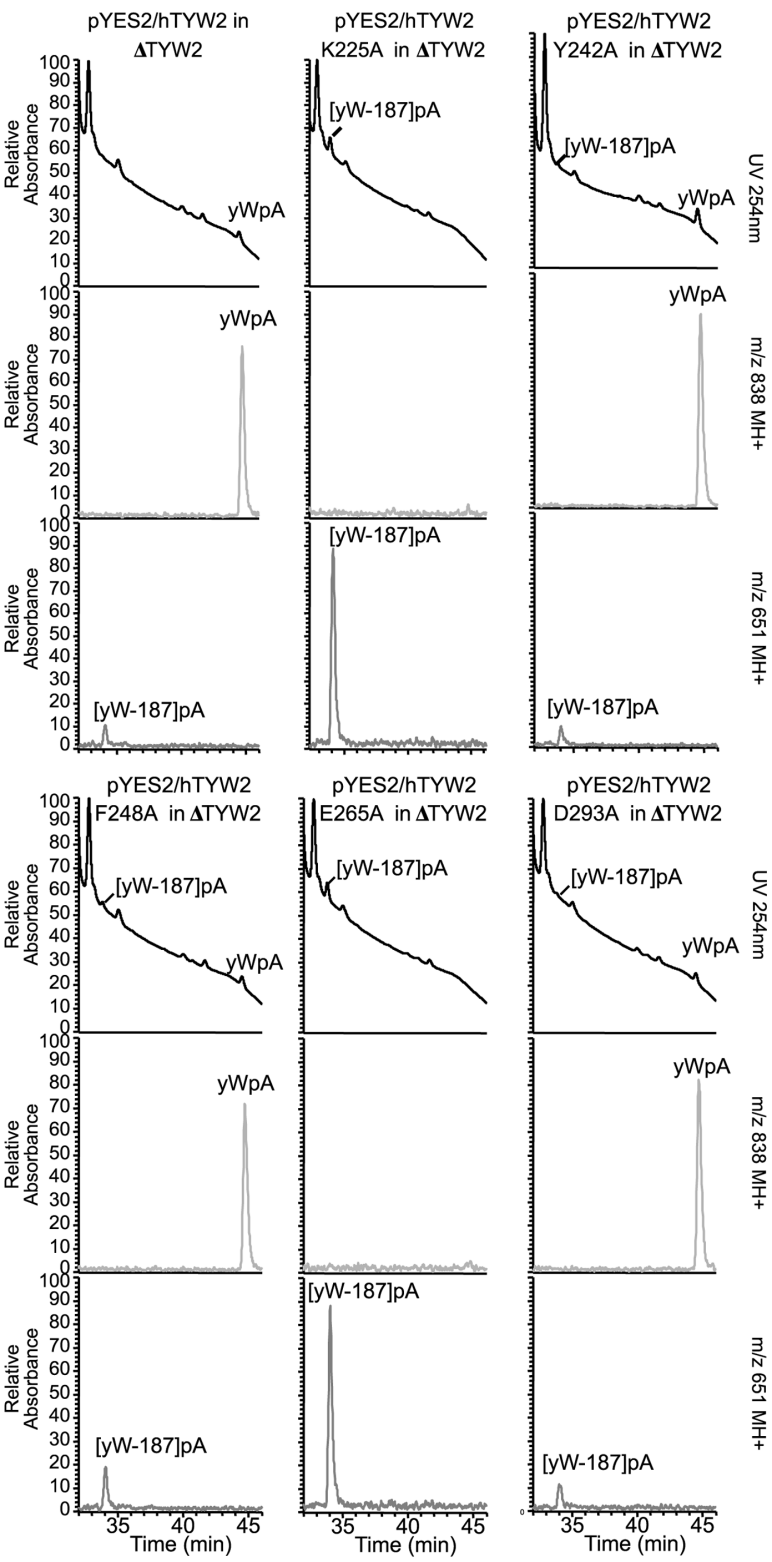
Mutagenesis of hTYW2 and evaluation of its effect on its enzymatic function in yW biosynthesis. Mutants, K225A, Y242A, F248A, E265A, and D293A were introduced into the hTYW2 gene by oligo-directed mutagenesis. These mutant protein expression constructs in the pYES2 vector were used to transform the yeast TYW2 deletion strain (ΔTYW2). All the mutant constructs expressed the TYW2 protein (not shown). LC/MS analysis of nuclease P1 digested tRNA-Phe obtained from the deletion strain ΔTYW2 transformed with wild type (top left) and the five mutant hTYW2 constructs in pYES2. For each transformant, the panels show UV at 254 nm (top), and mass chromatograms detecting MH+ (m/z838) of yWpA (middle), and MH+ (m/z 651) of [yW-187]pA (bottom) respectively.

**Figure 4:**
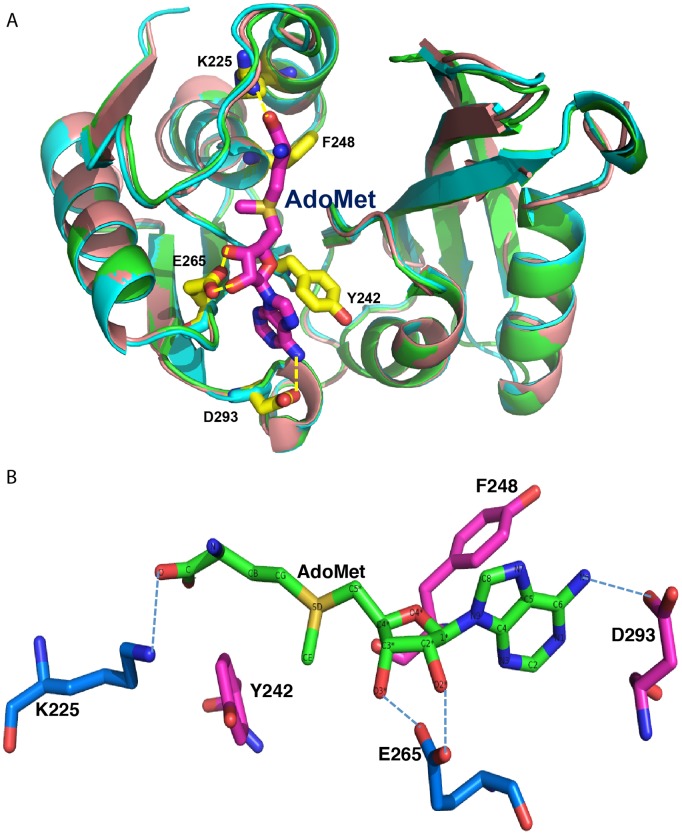
Superposition of modeled hTYW2 with the structure its archaeal homolog. A) Superposition of the modeled hTYW2 (in beige) with the crystal structures of *Pyrococcus horikoshii* TYW2 in the AdoMet bound (PDB-ID:3A25, in green) and unbound (PDB-ID:3K6R, in cyan) forms is shown. The figure was created using PyMOL visualization software (www.pymol.org). The strands are displayed as arrows. The AdoMet is shown as a stick model colored in pink with nitrogen atoms in dark blue and oxygen atoms in red. The five amino acid residues in the AdoMet binding pocket of hTYW2 that were chosen for mutagenesis are labeled, and the H-bonds formed by these residues with AdoMet are shown in yellow dashed lines. B) A view of the AdoMet binding pocket with only AdoMet and the residues chosen for mutagenesis. AdoMet is shown as a stick with the atoms labeled. H-bonding interaction of the residues with AdoMet are indicated by dashed-lines: E265 with the O2* and O3* of the ribose moiety, D293 with N6 of the Adenine ring, and K225 with the terminal oxygen of AdoMet.

We chose to evaluate the role that each of the five candidate amino acids play in yW biosynthesis by converting each to alanine: K225A, Y242A, F248A, E265A, and D293A. We generated the five mutant hTYW2 constructs in the yeast vector, pYES2, and evaluated their enzymatic activity in yeast by determining their ability to substitute the yeast homolog. We found mutations K225A and E265A eliminated the enzyme activity whereas mutants Y242A, F248A and D293A retained the enzyme activity, showing that K225 and E265 are critical residues for enzyme function (Figure 3). Figure 4, a superposition of modeled hTYW2 with the crystal structures of its archaeal homolog *Pyrococcus horikoshii* TYW2 (PDB-ID: 3K6R and 3A25, free protein and bound to AdoMet respectively) conveys how residues K225 and E265 interact with the acp donor AdoMet. E265 H-bonds with the O2* and O3* of the ribose moiety, and K225 H-bonds with the terminal oxygen of AdoMet.

### Mammary Tumor tRNA^Phe^ Contained Fully Modified yW Base

**Figure 5:**
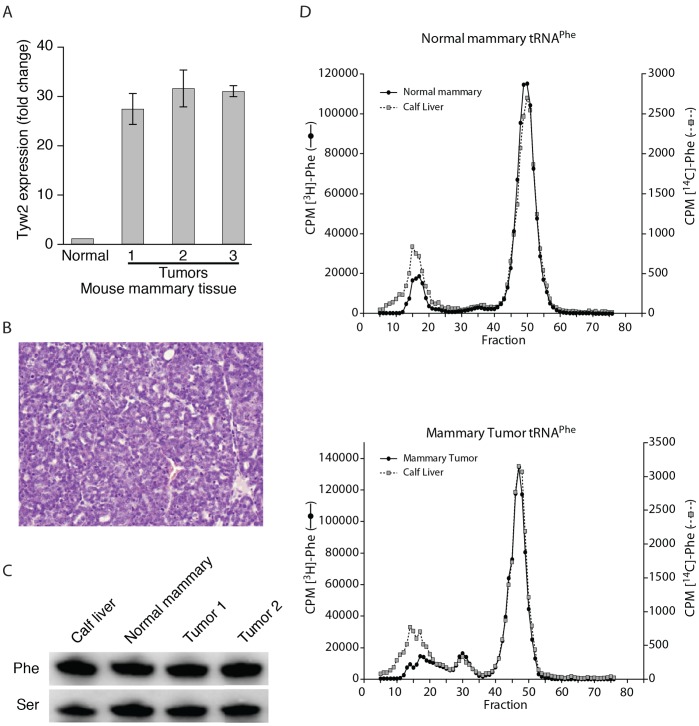
tRNA^Phe^ from normal and tumor mammary tissue. Mammary tumor tissue was obtained from mice heterozygous for the C3 (1)/SV40-T/t-antigen [C3 (1)/Tag] transgene in the FVB/N background. Expression of *Tyw2*, tumor histology, and quantitation of tRNA^Phe^ are described in A-C. (A) *Tyw2* expression in three mouse mammary tumor measured by RT-qPCR relative to *ß2M*. (B) Histology of paraffin-embedded C3(1)/Tag mouse mammary tumor showing adenocarcinoma. (C) Northern blot analysis of tRNA^Phe^ from C3(1)/Tag mouse mammary tumors, normal mouse mammary tissue from the same strain(FVB/N), and the calf liver control. The probe, Ser, refers to hybridization with a control probe for tRNA^Ser1^ [15]. (D) Elution profile from a RPC-5 column of Phe-tRNA^Phe^ from normal and tumor tissue. Isolation of tRNA, aminoacylation, and chromatography are as described in Materials and Methods. The profiles of [^3^H]-Phe tRNA from normal (upper panel) or tumor (lower panel) mouse mammary tissues are shown. [^14^C]-Phe tRNA from calf liver is included as an internal control in both the panels. A peak (around fraction 50) represents the tRNA^Phe^ with the fully modified yW base, and this is present in both the tRNAs from calf liver and the normal mouse mammary tissue (upper panel) and similarly from the tumor tissue (lower panel).

Since hTYW2 plays a role in the formation of yW, it was of interest to investigate whether the overexpression of hTYW2 observed in human breast tumors affected tRNA^Phe^ in mouse mammary tumor tissues. Eight individual tumor samples were obtained from three different types of transgenic mouse mammary tumor models, and tested for *Tyw2* expression. Each tumor showed increased *Tyw2* expression (Figure S3). The mouse mammary tumor model C3(1)/Tag [8] was selected because these tumors showed an average >20 fold increase in *Tyw2* gene expression (Figure 5A) as compared to the normal mammary tissue. The tumor histology was consistent with adenocarcinomas (Figure 5B) [8], [23]. To analyze the status of the yW base, total tRNA was extracted from each type of tissue and aminoacylated with [^3^H]phenylalanine and fractionated using reverse phase column chromatography (Figure 5D); the tumor analyzed here showed 31 fold higher expression of *Tyw2* compared to normal tissue. An internal control (calf liver [^14^C] tRNA^Phe^) was included in each experiment. The tRNA^Phe^ in both the normal mouse mammary tissue (top panel), and the tumor tissue (bottom panel) contained the fully modified wybutosine base. No quantitative differences in the levels of tRNA^Phe^ were observed in these tissues (Figure 5C), nor were there differences in many of the other modified bases found in tRNA^Phe^ (data not shown). Thus, the yW base modification and expression level of tRNA^Phe^ appeared unaffected in mouse mammary tumors.

## Discussion

We have established that human TYW2 encodes the enzyme that is required for the posttranscriptional modification of the base G to yW in tRNA^Phe^ (Figure 1). The human TYW2 gene could substitute for its yeast counterpart in providing the enzyme needed for the catalysis of the third step in the conversion of G37 to yW, and thus indicating that it plays a similar role in the posttranscriptional modification of tRNA^Phe^ in humans. Even though a large number of post-transcriptional base modifications in tRNAs have been well characterized, including those from mammalian tissues, the enzymes that catalyze many of these modifications are yet unclear, particularly in human or other mammalian tissues. yW modification is the most complex modification in tRNA, and an enzyme component of this pathway in humans has now been characterized.

Earlier, we reported amplification and overexpression of TYW2 in breast cancer and now we have found that it catalyzes a step in the modification of G(37) to yW base in tRNA^Phe^. Though the importance of this enzymatic function in tumorigenesis is not immediately apparent, it was of interest to note that over 30 years ago tRNA^Phe^ from mouse neuroblastoma was shown to be undermodified where the yW base is replaced with methyl-1-guanosine [5]. The undermodification of the yW base in tRNA^Phe^ allowed for retroviral –1 frameshifting during translation in mammalian cells [6] and recently, using Xenopus oocyte system, it has been demonstrated that the hypomodified tRNA^Phe^ not only enhances but is required for frameshifting [24]. Therefore, we determined whether overexpression of the hTYW2 disrupted the wybutosine pathway in the mammary tumor cells. We found that mouse mammary tumor models overexpressed Tyw2, but the tumor tissue contained the fully modified yW base and did not show quantitative differences in the levels of tRNA^Phe^ (Figure 5).

Abnormalities in the modified bases in tRNAs have been described in tumors [25], implying the critical roles of modifying enzymes in cancer. An increase in the number of dihydrouridine (D) base in tRNA^Phe^ from tumors was reported in 1978 [25]. Over three decades later, dihydrouridine synthase (hDUS) was found to be overexpressed in lung cancer, and its expression levels correlated with the aggressiveness of the tumors and, thus, the patients’ survival [26]. The tRNA methyltransferase, Trm9, was linked to the regulation of DNA damage response proteins in yeast [27]. Trm9 is required for the posttranscriptional modification of uridine, the 34^th^ base at the wobble position of the anticodon in tRNA^Arg^. In that study, deletion of Trm9 and the associated failure in modification resulted in tRNA^Arg^ being ineffective when reading the codons enriched in certain key DNA damage response proteins, decreasing the efficiency of their translation [27]. Interestingly, the human homolog of Trm9 is located on chromosome 8 at 8p22, which is an oft deleted region in colorectal cancer [28], and microcell transfer studies implicate that 8p22 may harbor a putative tumor suppressor [29]. Mutations in Dyskerin (DKC1), the human homolog of an rRNA modifying pseudouridine synthase, leads to dyskeratosis congenita, a premature aging syndrome with progressive bone marrow failure syndrome with predisposition to malignancy [30]. However, no correlation has yet been made to the rRNA modification and the inactivation of DKC1.

The tRNA modification enzymes described above, *via* their ability to alter modifications in tRNAs and translational regulation, play a key role in cancer or cell biology in general. However, we conclude that overexpression of hTYW2 in mammary cancer may not adversely affect the yW modification. It is possible that this overexpression may adversely affect another biological function that is mediated by this enzyme activity. Alternatively, human TYW2 may have a yet unknown additional function that may have a role in tumorigenesis. In yeast, TYW2 was found to interact with SUP35 protein [31]. The mammalian homolog of the yeast SUP35 is a cell cycle protein, eRF3/GSTP1. It is tempting to speculate that further exploration of the TYW2 interacting proteins in mammalian cells might provide clues to its role in tumorigenesis. Our previous report on the amplification and overexpression of this gene in breast cancer is the only report on mammalian TYW2. This was apparent when we performed network reconstruction (protein-protein interactions) using MetaCore (GeneGo, Inc, St. Joseph, MI) in an effort to understand the pathways TYW2 is involved with (data not shown). However, from MetaCore analysis it appears that TYW2 is a target of transcriptional regulation based on chromatin immunoprecipitation studies for proteins cyclinD1 [32], ESR1 [33], and HNF4- α and HNF6 [34]. Confirming and extending these observations as to whether these interactions result in altered expression of TYW2 might open up avenues to explore biological consequence of overexpression of TYW2.

Based on structure-guided sequence alignment of hTYW2 with its homologs, we chose to evaluate the importance of five amino acids by site-directed mutagenesis. Two of the five mutants, K225A and E265A, lacked enzyme activity, showing that K225 and E265 are critical residues for the acp transferase activity of hTYW2 enzyme. A comparison of the positions of these mutations on the structure shows that the glutamic acid (E265) residue forms a H-bond with the ribose moiety, which is involved in catalysis in the known methyltransferases [35]. The lysine (K225) residue forms H-bond with the terminal oxygen, and is positioned close to the propyl group being transferred in this reaction (Figure 1 and 4). Thus, for transfer of the acp group as opposed to a methyl group, the residues that are important are those that hydrogen bond with the terminal oxygen, and O2* and O3* of the ribose moiety. This is consistent with the mutagenesis of the corresponding residues in archaeal TYW2, E155A and R116A, which resulted in nearly eliminating the enzyme activity [20]. Structural and functional conservation of human TYW2 with its evolutionarily distant neighbor archaeal TYW2 is clearly apparent from modeling and mutagenesis studies.

## Supporting Information

Figure S1 **Single-Linkage clustering tree.** The tree was generated using CDTree tool (http://www.ncbi.nlm.nih.gov/Structure/cdtree/cdtree.shtml). There are three clusters corresponding to archaea, mammals and fungi. The PYRHO (archaea), HUMAN and YEAST TYW2 sequences are indicated by a star. The three clusters belong to three protein homeomorphic families (homeomorphic here indicates that the proteins that belong to a family have similar lengths and domain architectures). Representative sequences from these families, PIRSF006525 (archaea), PIRSF038667 (mammals) and PIRSF038972 (fungi) were used. Single-linkage clustering creates protein clusters with the restriction that the sequence of a protein recruited to a given cluster aligns with the recruiting sequence over at least 85% percent of both sequences. Initially, the aligned sequences must share 100% identity. Thereafter, the identity criterion is iteratively decremented by one and clustering is repeated. Trees are produced by examination of clusters at each iteration. Since this is a clustering tree, no bootstrap values are provided. Six sequences represent the PDB IDs, and are derived from crystal structures of *Pyrococcus horikoshii* TYW2 (PhTYW2) and *Methanocaldococcus jannaschii* TRM5 (MjTRM5): 3K6R A (PhTYW2), 3A25 A (PhTYW2 with AdoMet), 2YX1 A (MjTRM5), 2YX1 B (MjTRM5), 2ZZM A (MjTRM5 with tRNA^Leu^), 2ZZN A (MjTRM5 with tRNA^Cys^). Other sequences are labeled using UniProtKB accessions (www.uniprot.org), and the complete names of the species are provided in Table S1.
(TIF)

Figure S2 **Structure-guided alignment of hTYW2 protein families and their homologs.** The alignment shows all representative sequences that belong to three families as classified by PIR (pir.georgetown.edu) named PIRSFs. The TYW2 members belong to PIRSF006525(archaea), PIRSF038972(fungi) and PIRSF038667(mammals). The alignment was created using the Cn3d tool. The residues are colored based on the level of conservation with highly conserved residues in red to not conserved residues in blue. The residues in lower case letters indicate regions of no conservation. This alignment includes the transferase domain (amino acids 118–336) and extends to the c-terminus (amino acid 448) of hTYW2. The positions of the five residues in TYW2 HUMAN chosen for carrying out mutagenesis are shadowed in gray (K225, Y242, F248, E265 and D293). The residues in TYW2 PYRHO that were analyzed by mutagenesis (taken from Umitsu et al Proc Natl Acad Sci U S A 106: 15616–15621) are indicated by circles on top, and those resulting in the severe inactivation of the enzyme activity (>90%) are shown with the filled circles (taken from Umitsu et al Proc Natl Acad Sci U S A 106: 15616–15621). The sequences are labeled using UniprotKB accessions (www.uniprot.org), and the complete names of the species are provided in Table S1.
(TIF)

Figure S3 **Quantitative** ***Tyw2*** **expression analysis in RNA from multiple mouse mammary tumor tissues.** RT-qPCR was performed on RNA from eight individual tumor samples. The number of individual tumors and the mammary tumor model they were derived from is indicated in the X-axis. These tumors are from three different mouse mammary tumor models generated by transgenic expression of SV40 -T/t antigen [C3(1)/Tag], Polyoma middle T oncogene [MMTV-PymT] and Her2/neu oncogene [MMTV-Her2/neu]. C3(1)/Tag tumors are from different lineages than those presented in Figure 5. The fold change in Tyw2 gene expression (relative to that from “Normal” FVB/N strain) is shown in the Y-axis. The expression of housekeeping gene b2M was used as an internal control. Each bar represents the mean and SD of measurements in triplicates.
(TIF)

Table S1 **Complete names of the organisms.** The names of the organisms in the UniProt Accessions used to generate Figures S1 and S2 are expanded to provide complete names.
(XLSX)
