# Supplementary figures and images for: Structure-Function Analysis of Human TYW2 Enzyme Required for the Biosynthesis of a Highly Modified Wybutosine (yW) Base in Phenylalanine-tRNA
Source: PLoS One. 2012 Jun 28;7(6):e39297. doi: 10.1371/journal.pone.0039297 (PMC3386263; doi:10.1371/journal.pone.0039297)

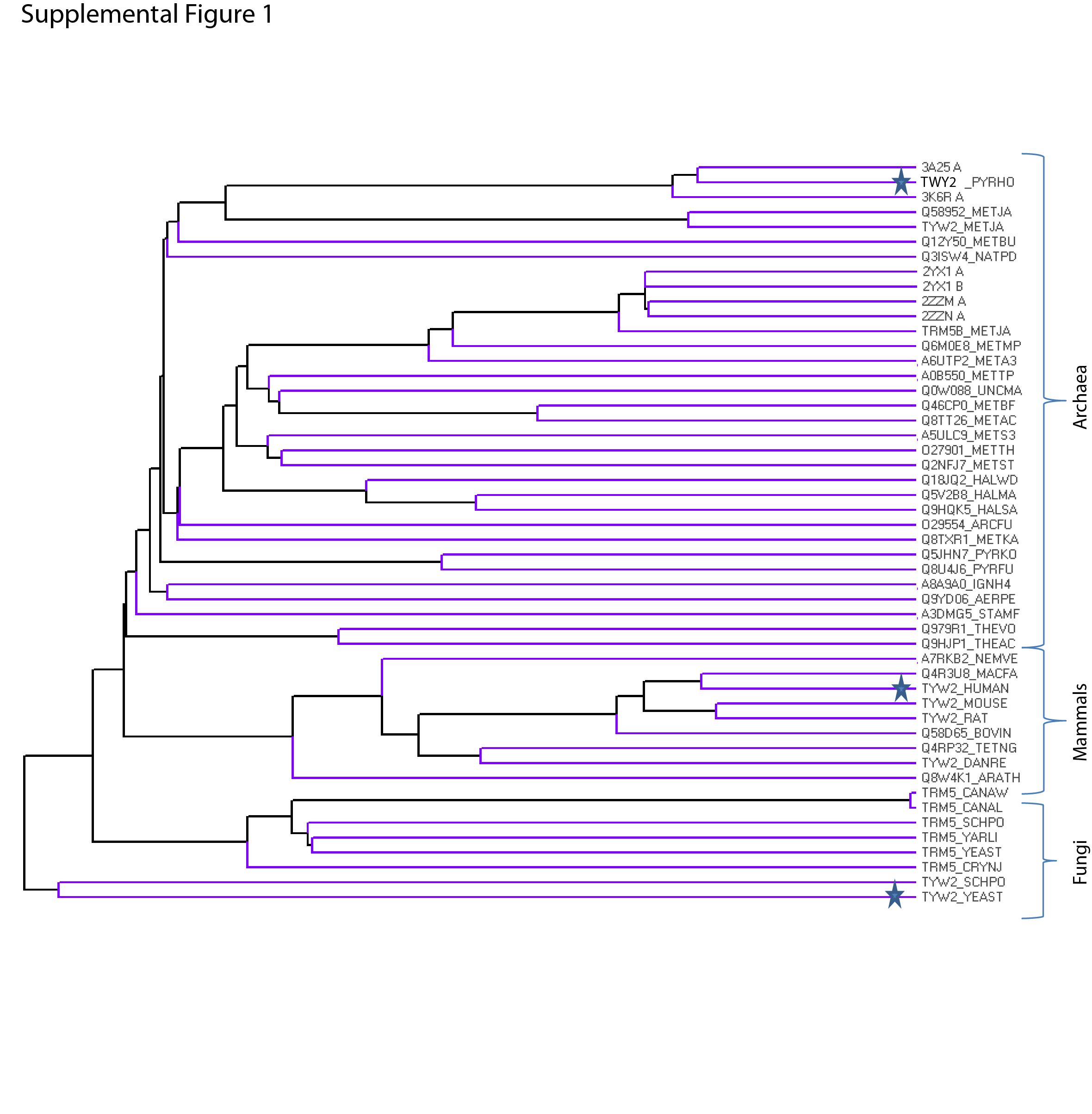

Supplement: Figure S1 — Single-Linkage clustering tree. The tree was generated using CDTree tool (http://www.ncbi.nlm.nih.gov/Structure/cdtree/cdtree.shtml). There are three clusters corresponding to archaea, mammals and fungi. The PYRHO (archaea), HUMAN and YEAST TYW2 sequences are indicated by a star. The three clusters belong to three protein homeomorphic families (homeomorphic here indicates that the proteins that belong to a family have similar lengths and domain architectures). Representative sequences from these families, PIRSF006525 (archaea), PIRSF038667 (mammals) and PIRSF038972 (fungi) were used. Single-linkage clustering creates protein clusters with the restriction that the sequence of a protein recruited to a given cluster aligns with the recruiting sequence over at least 85% percent of both sequences. Initially, the aligned sequences must share 100% identity. Thereafter, the identity criterion is iteratively decremented by one and clustering is repeated. Trees are produced by examination of clusters at each iteration. Since this is a clustering tree, no bootstrap values are provided. Six sequences represent the PDB IDs, and are derived from crystal structures of Pyrococcus horikoshii TYW2 (PhTYW2) and Methanocaldococcus jannaschii TRM5 (MjTRM5): 3K6R A (PhTYW2), 3A25 A (PhTYW2 with AdoMet), 2YX1 A (MjTRM5), 2YX1 B (MjTRM5), 2ZZM A (MjTRM5 with tRNALeu), 2ZZN A (MjTRM5 with tRNACys). Other sequences are labeled using UniProtKB accessions (www.uniprot.org), and the complete names of the species are provided in Table S1. (TIF) [file pone.0039297.s001.tif]

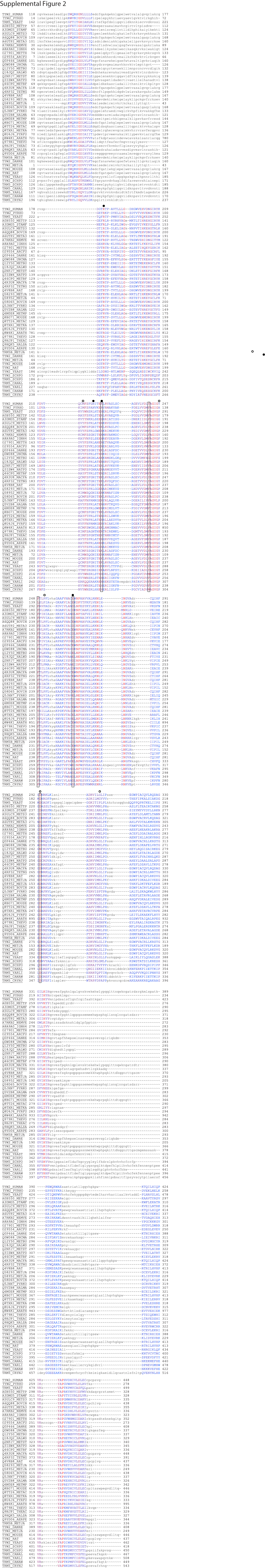

Supplement: Figure S2 — Structure-guided alignment of hTYW2 protein families and their homologs. The alignment shows all representative sequences that belong to three families as classified by PIR (pir.georgetown.edu) named PIRSFs. The TYW2 members belong to PIRSF006525(archaea), PIRSF038972(fungi) and PIRSF038667(mammals). The alignment was created using the Cn3d tool. The residues are colored based on the level of conservation with highly conserved residues in red to not conserved residues in blue. The residues in lower case letters indicate regions of no conservation. This alignment includes the transferase domain (amino acids 118–336) and extends to the c-terminus (amino acid 448) of hTYW2. The positions of the five residues in TYW2 HUMAN chosen for carrying out mutagenesis are shadowed in gray (K225, Y242, F248, E265 and D293). The residues in TYW2 PYRHO that were analyzed by mutagenesis (taken from Umitsu et al Proc Natl Acad Sci U S A 106: 15616–15621) are indicated by circles on top, and those resulting in the severe inactivation of the enzyme activity (>90%) are shown with the filled circles (taken from Umitsu et al Proc Natl Acad Sci U S A 106: 15616–15621). The sequences are labeled using UniprotKB accessions (www.uniprot.org), and the complete names of the species are provided in Table S1. (TIF) [file pone.0039297.s002.tif]

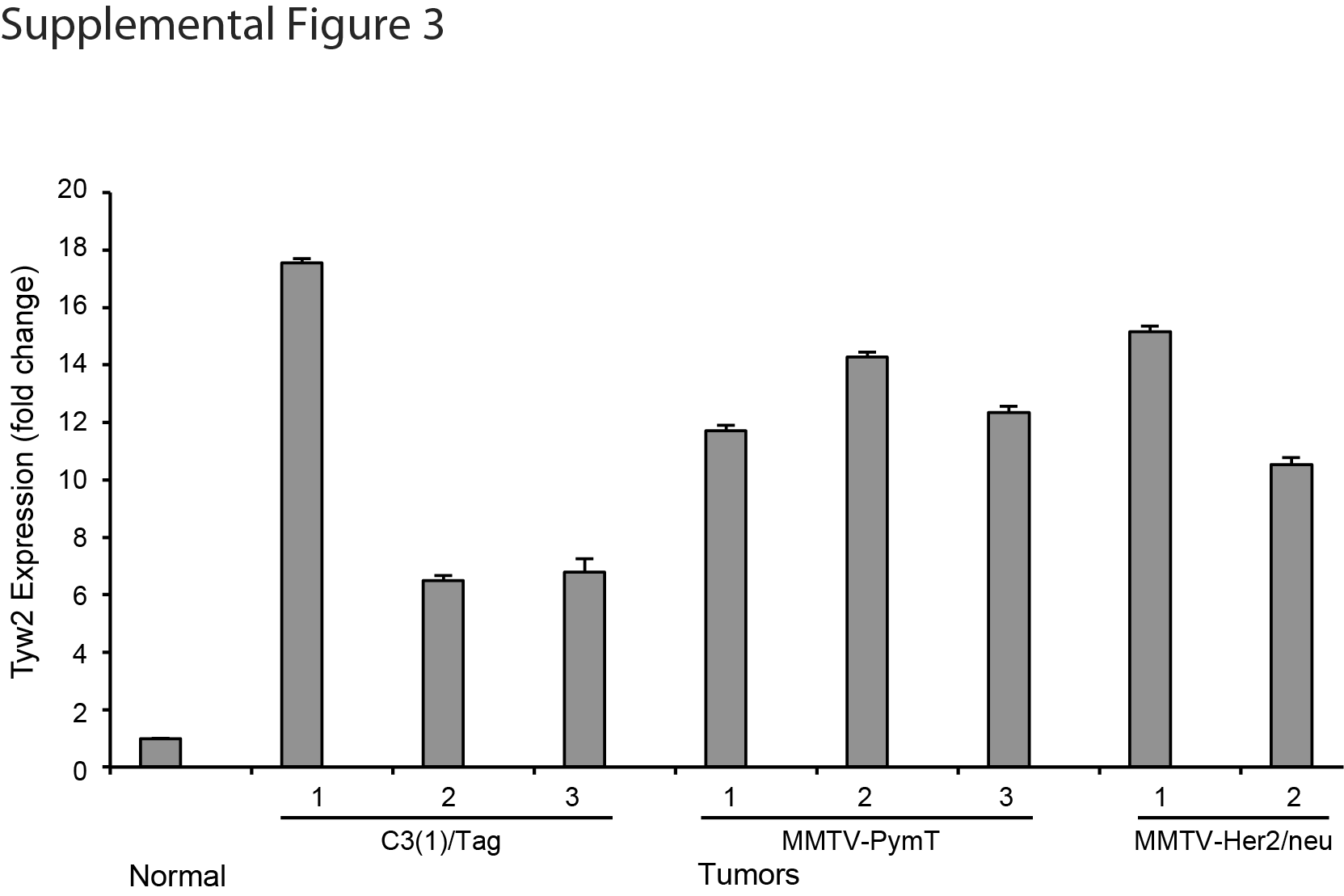

Supplement: Figure S3 — Quantitative Tyw2 expression analysis in RNA from multiple mouse mammary tumor tissues. RT-qPCR was performed on RNA from eight individual tumor samples. The number of individual tumors and the mammary tumor model they were derived from is indicated in the X-axis. These tumors are from three different mouse mammary tumor models generated by transgenic expression of SV40 -T/t antigen [C3(1)/Tag], Polyoma middle T oncogene [MMTV-PymT] and Her2/neu oncogene [MMTV-Her2/neu]. C3(1)/Tag tumors are from different lineages than those presented in Figure 5. The fold change in Tyw2 gene expression (relative to that from “Normal” FVB/N strain) is shown in the Y-axis. The expression of housekeeping gene b2M was used as an internal control. Each bar represents the mean and SD of measurements in triplicates. (TIF) [file pone.0039297.s003.tif]
